# Translation, cultural adaptation, and validation of the Nurse Self-Concept Questionnaire (NSCQ) for Portuguese nursing students

**DOI:** 10.1186/s12912-024-02031-7

**Published:** 2024-06-21

**Authors:** Inês F. Almeida, Rafael A. Bernardes, Liliana B. Sousa, Paulo Santos-Costa, Filipa Ventura, Amorim Rosa

**Affiliations:** 1 Health Sciences Research Unit: Nursing, Nursing School of Coimbra, Coimbra, Portugal; 2 The Nursing Research, Innovation and Development Centre of Lisbon (CIDNUR), Nursing School of Lisbon (ESEL), Lisboa, Portugal

**Keywords:** Nursing students, Nursing, Professional self-concept, Mental health, Psychometrics

## Abstract

**Background:**

The professional self-concept of nursing students significantly influences their attitude and identity within the profession, ultimately impacting their mental health and overall well-being. Recent evidence underscores the importance of assessing students’ professional self-concept to prevent adverse outcomes such as burnout and stress. Since there are currently no validated instruments available in Portugal for this purpose, our objective was to translate, adapt, and validate the Nurse Self-Concept Questionnaire (NSCQ) with nursing students in Portugal.

**Methods:**

A two-phase research study with a non-probabilistic sample of 216 undergraduate nursing students, using the *Qualtrics*^*XM*^ electronic platform for data collection. An exploratory factor analysis was performed to verify the validity of the theoretical construct and its internal consistency. Cronbach’s alpha was calculated, and a confirmatory factor analysis was performed to assess the model fit.

**Results:**

The final instrument, designated as Questionário de Autoconceito dos/as Enfermeiros/as (Pt – NSCQ), is composed of 24 items distributed across five dimensions: “General self-concept”, “Staff relations”, “Leadership”, “Communication-care” and “Knowledge”, which explain 67.71% of the total variance. All dimensions and the global scale revealed good internal consistency values, ranging from 0.775 to 0.927. The resulting factorial structure is coherent with the theoretical framework.

**Conclusion:**

The Pt – NSCQ proved to be a valid and reliable instrument to assess Portuguese nursing students’ professional self-concept. Future studies should be carried out on larger samples and different educational contexts, aligned with the importance to ensure the continuity of the psychometric analysis of the instrument.

**Supplementary Information:**

The online version contains supplementary material available at 10.1186/s12912-024-02031-7.

## Background

Nurses are a vital and significant professional group in healthcare, responsible for ensuring efficient, safe, and sustainable care delivery [1, 2]. In the nursing profession, the professional self-concept is particularly relevant [3] and is understood as a prerequisite for the vocational and academic development of the professional identity [4–6].

The professional self-concept is multifaceted, encompassing individuals’ perceptions and comprehension of themselves within their professional roles. It influences their thought processes, the evolution of their roles, and ultimately, their professional conduct and performance [7]. ‘Professional self-concept’ in nursing encompasses an understanding of personal identity in relation to one’s professional role, and guides communication, thoughts, and behavior in practice [8, 9]. The concept significantly influences nurses’ capacity to deliver high-quality care and achieve positive health outcomes [10]. A robust professional self-concept enhances nurses’ adaptation to real-world challenges, fosters their accountability, and supports evidence-based practice [11–13]. Evidence supports that nurses with strong professional self-concept experience higher levels of self-esteem, self-efficacy, and overall well-being, contributing positively to their mental health and coping with professional and daily life challenges [14]. Conversely, a diminished professional self-concept may lead to burnout and a negative view of the profession [3, 15, 16]. Such reality is especially significant during critical scenarios such as pandemics or geopolitical conflicts [15].

The development of professional self-concept is the result of the interaction with other professionals, and its construction begins already during the nursing training [17], following a dynamic process that is influenced by age, cognitive development and other individual and environmental factors [18]. Whereby nursing education is necessary and can cultivate students’ strong professional self-concept [19]. The professional self-concept significantly shapes various aspects of nursing students’ well-being and development. It plays a crucial role in mental health, academic performance, and the development of professional values [18, 20, 21]. A high professional self-concept is associated with positive outcomes, such as improved clinical performance, reduced burnout, lower academic stress, increased satisfaction, and higher academic self-efficacy [22–25].

Since there are currently no validated instruments available in Portugal for the purpose of assessing the professional self-concept in nursing, the present study aims to translate, culturally adapt, and validate the “Nurse’s Self-concept Questionnaire” (NSCQ) to European Portuguese nursing students.

## Methods

### Study design, participants and setting

To verify the validity and reliability of the NSCQ, the cultural adaptation and psychometric properties of the tool, this study was divided into two phases involving the translation and cultural adaptation of the NSCQ from English to European Portuguese, and the cross-sectional psychometric evaluation of the NSCQ. This study was conducted at the Nursing School at the Centre Region of Coimbra from August 2022 to September 2023.

The distribution of the NSCQ, was also preceded by a sociodemographic questionnaire, which entails various aspects, including gender, age, marital status, higher education institution, year of enrolment in the Nursing degree program, clinical internship details, previous degree in the health field, retention, and therapy involving psychotropic drugs.

#### Phase I: Translation and cultural adaptation

In compliance with the cross-cultural adaptation guidelines suggested by Beato et al. (2000), the original NSCQ scale items in English were translated into European Portuguese [26]. The translation process was as follows:

1. Forward translation: Two independent translators, one with knowledge of the core subject (professional self-concept) and the other without it, translated the original instrument into European Portuguese (T1 and T2, respectively). Both translators had previous experience in the translation of assessment instruments.
2. Synthesis: T1 and T2 were carefully compared, including title, items, and response scale options. The translators and the research team developed a synthesized version (T1-2).
3. Back translation: T1-2 was meticulously translated back into English by two proficient bilingual translators, entirely independent of the study and unfamiliar with the scale. The back-translated version (BT1-2) was thoroughly compared with the original to ensure accuracy through meticulous proofreading.
4. Expert committee review: An expert committee, comprising 10 healthcare professionals from diverse backgrounds (teachers, nurses, researchers, and psychologists), with no prior knowledge of the NSCQ, participated in a Delphi Panel methodology [27] through the *Qualtrics*^*XM*^ platform (Washington, United States). The inclusion criteria were: (i) health professionals; (ii) teachers at nursing schools or research fellows in health sciences; (iii) have no previous contact with the assessment tool under study. Each participant received the translated items and compared them with the original aiming for consensus on any discrepancies. The Portuguese exploratory version of the NSCQ was considered valid when an agreement of 80% or higher was reached, with scores between 3 and 5 on a scale of 1 to 5, and an overall score equal to or higher than 40 points (4 × 10), corresponding to an average of 4 points per item [28].
5. The exploratory version of the Portuguese NSCQ was tested through a pilot study using a non-probabilistic snowball sampling method. According to Beaton et al. (2000), both the meanings of the items and the answers should be explored and, this can be verified through the presence of missing values in the answers to the evaluation instrument [26]. The sample consisted of 20 undergraduate nursing students (i.e., five for each year of the degree, which is four years in the study context) who met the following inclusion criteria: (i) were students enrolled in the bachelor’s degree in nursing; (ii) with or without any previous clinical experience in real contexts. Participants were excluded if they: (i) were undergoing pharmacological treatment with psychotropic drugs at the time; (ii) had worked in other health professions (e.g. medical assistants, audiologists); (iii) had previous contact with the Pt – NSCQ/NSCQ.
6. The author of the original instrument was continuously engaged from the beginning and through all research steps, having validated all the changes made to the instrument as well as the final version.

#### Phase II: Validation study

Data collection for evaluation of the psychometric properties was undertaken on January 2023, using an online version of the *“Questionário de Autoconceito dos/as Enfermeiros/as”* (a.k.a., Pt – NSCQ following the experts’ consensus) created on *Qualtrics*^*XM*^. A non-probabilistic snowball strategy was used through email dissemination to 12 public and private Portuguese nursing schools (seven from the centre of Portugal, three in the north and two in the Lisbon metropolitan region) and students to recruit participants to the study, following the same inclusion and exclusion criteria as those previously established for step 5 (phase I). A minimum number of five participants for each of the 36 items of the Pt – NSCQ was considered to ensure the preservation of the factorial structure during data analysis [29, 30]. Therefore, we aimed for a minimum sample of 180 participants.

The psychometric properties of the Pt – NSCQ were assessed, namely the validity and reliability parameters. Regarding construct validity, Exploratory Factor Analysis (EFA) was conducted to identify the underlying factors of the nursing self-concept, and Confirmatory Factor Analysis (CFA) was used to evaluate the model fit. Internal consistency (cronbach alphas) and item-total correlations were calculated for reliability.

### Original instrument description and sociodemographic data sheet

The NSCQ was originally developed and validated in Australia by Cowin (2001) to evaluate the professional self-concept in nursing. It comprises 36 items evenly distributed across 6 dimensions (i.e., General self-concept; Care; Staff Relations; Communication; Knowledge; Leadership) [31]. The original questionnaire employed an 8-point Likert-type scale, where respondents rated each item from 1 (Definitely false) to 8 (Definitely true). The resulting score ranges from 36 to 288, with higher scores indicating better professional self-concept. The six-factor structure was validated, with 72.9% of total variance explained. The internal consistency values for the six dimensions of the instrument were considered good to very good, ranging from 0.83 (knowledge dimension) to 0.93 (general and leadership dimensions), indicating good reliability of the measurement [31].

The NSCQ has been adapted for use in Turkey [16], China [32], and Taiwan [33], and validated in samples of nurses and/or nursing students. It is noteworthy that tool has consistently demonstrated strong psychometric properties regardless of when applied across the context of the above-mentioned countries.

### Statistical analyses

The data analysis was carried out using IBM Corporation’s Statistical Package for the Social Sciences (SPSS) software version 24.0 in Somers, New York, USA. Descriptive statistics were employed, including measures of central tendency and dispersion. Quantitative data were summarized using Mean (M), standard deviation (SD), minimum (Min.), and maximum (Max.), while nominal and ordinal data were presented as absolute (n) and percentage (%) frequencies. A statistically significant difference was considered when *p* < 0.05.

Data suitability for the EFA was determined by performing Bartlett’s Test of Sphericity (BST) and the Kaiser-Meyer-Olkin (KMO) test. A significant BST value (*p* < 0.05) indicated appropriateness for factor analysis [34, 35]. The KMO test assessed sample size sufficiency. A KMO value closer to 1.0 indicated better suitability, below 0.50 was unacceptable, 0.50 to 0.60 was acceptable but poor, 0.60 to 0.70 was average, 0.80 to 0.90 was good, and above 0.90 was excellent [34]. The principal component analysis and varimax orthogonal rotation aim to identify a factor solution consistent with the theoretical framework, assuming no significant inter-factor relationships. Retention criteria for factors and items from the Pt – NSCQ version were based on Eigenvalues greater than one, commonality coefficients (*h²*) above 0.40, factor loadings of at least 0.50, and differential loadings of 0.20 or greater compared to other factors [35, 36].

The AMOS 24 software (AMOS Development Corp., Crawford, FL, USA) was used for the CFA. The underlying factors of the adapted cultural profile was assessed via CFA. The following indices were used to assess the goodness-of-ft of the data: Comparative Fit Index (CFI) with a cut-of value of CFI ≥ 0.90, the normed χ2 with a cut-of value of normed *χ2/df* ≤ 3, Root Mean Squared Error of Approximation (RMSEA) with a cut-off value of RMSEA ≤ 0.08 [37]. The Root Mean Square residual (RMR) was also determined, which must be as close to 0 as possible [38], as well as the Goodness of Fit Index (GFI) with a cut-off value of GFI ≥ 0.90 [39].

To assess reliability, Cronbach’s Alpha, and the ‘alpha if item deleted’ coefficient was determined. Values of Cronbach’s Alpha between 0.70 and 0.79 indicated acceptable internal consistency, 0.80 to 0.89 showed good internal consistency, and above 0.90 indicated excellent internal consistency [40, 41]. Also, Item-total correlation and Cronbach’s alpha coefficients were used as internal consistency estimates.

### Ethical considerations

The study was approved by the Ethics Committee of the Health Sciences Research Unit: Nursing. at the Nursing School of Coimbra (references number P884_06_2022 and P925_11_2022). Ethical and legal standards were strictly followed throughout the study. The research was carried out in strict compliance with the Declaration of Helsinki.

Permission to translate, adapt, and validate the instrument into European Portuguese was sought via email from the original author, and mutual agreement was achieved.

All participants took part in the study on a voluntary basis. Before their involvement, they were fully informed about the purpose and nature of the research, and they provided a signed informed consent to participate in the study.

## Results

### Translation and cultural adaptation

The translation process was conducted systematically, yielding no noteworthy discrepancies between the translated versions. This led to a consensus synthesis (T1-2). The consensus was corroborated by two independent translators, who advocated for streamlining the Likert-type response scale from 8 to 6 points. Their proposal involved merging point 3 (Mostly false) with point 4 (More false than true) and point 5 (More true than false) with point 6 (Mostly true), due to their perceived similarity. The research team discussed this change exposing and explaining to the author of the creation and validation of the original Instrument and approved this change during the study’s step 4 (Phase I).

In the back-translation step, a notable degree of similarity and consensus emerged between the two versions, culminating in the creation of the synthesis of the back translations. This version was used during the initial Delphi panel, facilitating comprehension and analysis for the experts.

Among the participants of the Delphi panel, six were female (60.0%), aged between 34 and 68 (46.80 ± 9.56). Regarding their academic qualifications, four (40.0%) participants had a master’s degree, five (50.0%) had a PhD and, one (10.0%) had a post-doctorate degree. Seven experts (70.0%) had experience supervising undergraduate nursing students in clinical teaching contexts. It should also be noted that four (40.0%) were teachers in the nursing undergraduate programme; three (30.0%) were nursing researchers; one (10.0%) was a psychologist working in research contexts; and two (20.0%) were specialist nurses working in a clinical context. The participants had between 12 and 35 (21.20 ± 7.67) years of professional experience.

During the first Delphi round, the name of the instrument and specific items (i.e., 1, 10, 14, 17, 18, 24, 35), with item 1 belonging to the Care dimension, items 10, 14 and 35 to the Knowledge dimension, item 17 to the Leadership dimension, item 18 to the Nurse General Self-Concept and item 24 to Staff Relations, failed to attain the consensus level of at least 80%. A second round of the panel was therefore conducted which included their amendment according to the experts’ suggestions. In the second round, consensus was successfully reached for all items and the instrument’s name, aligning with the predefined criteria.

In the pilot study, the comprehensibility of the instrument was also assessed, and an open space was left at the end of the instrument for comments on completion and interpretation. There were no missing responses to the items.

Participants of the pilot study were 20 nursing students between 18 and 22 (19.65 ± 1.35) years old, and the majority were female (75.0%). Around 35% were in the final year of their undergraduate degree, 31% in the third year, 17% in the second year and 15% in the first year.

### Psychometric validation

A total of 219 responses were collected for psychometric assessment purposes. Three were excluded due to the absolute unreliability of the answers [42], leading to a final sample of 216 participants. Students’ ages ranged from 18 to 45, with a mean age of 21.59 ± 4.63 years. Most of the respondents were female (82.4%) and single (95.4%). Academically, 77 (35.6%) were enrolled in the fourth and last year of the undergraduate degree, 68 (31.5%) were enrolled in the third year, 37 (17.1%) were enrolled in the second year, and 34 (15.7%) were freshmen.

The prerequisites for carrying out the EFA were verified, with the KMO sample adequacy measure attaining a value of 0.926. The BTS results also indicate that the data is suitable for carrying out factor analysis (*x²* = 5045.15; *p* = 0.000).

Principal component analysis with varimax rotation was performed and 3 extractions were conducted (Table 1). The first extraction, made with the initial 36-item instrument, resulted in a solution with eigenvalues between 13.905 and 1.005, with a total explained variance of 64.22%. Eight items (i.e., 2, 4, 7, 19, 23, 25, 29 and 34), with item 2 referring to staff relations, items 4, 19 and 25 to the knowledge dimension, item 7 to communication and items 23, 29 and 34 to care, were deleted as their factor loadings were below 0.50 and had differential factor loadings of similar magnitudes in more than one dimension [35, 36].

The second extraction resulted in a solution with five domains, which explained 65.61% of the total variance. However, four items (i.e., 10, 17, 30 and 36), with item 10 belonging to knowledge, item 17 to leadership and items 30 and 36 to communication, were deleted as their factor loadings were below 0.50 and had differential factor loadings of similar magnitudes in more than one dimension.

In the third and final extraction (Supplementary file 1), the remaining 24 items were kept, with good factor loadings, and 67.71% of the total variance explained. The communality values ranged from 0.479 to 0.853, meeting the criteria for retaining the five factors.

**Table 1:** Matrix of components extracted from the PCA, Eingvalues and explained variance (*n* = 216)

Items	Dimensions/factors					Corrected total itemised correlation	α if the item is deleted	h^2^
	General self-concept	Staff relations	Leadership	Care-communication	Knowledge			
12.	0.873					0.697	0.922	0.853
6.	0.860					0.624	0.923	0.793
3.	0.845					0.626	0.923	0.782
16.	0.815					0.642	0.923	0.740
27.	0.706					0.635	0.923	0.616
18.	0.696					0.627	0.923	0.632
31.	0.641					0.582	0.924	0.664
11.		0.729				0.529	0.925	0.639
9.		0.724				0.565	0.924	0.635
15.		0.705				0.565	0.924	0.667
32.		0.670				0.613	0.924	0.634
24.		0.664				0.618	0.924	0.638
28.			0.779			0.640	0.923	0.763
5.			0.763			0.444	0.928	0.622
8.			0.739			0.404	0.927	0.643
22.			0.736			0.558	0.924	0.677
33.			0.700			0.572	0.924	0.688
20.				0.758		0.581	0.924	0.717
26.				0.675		0.548	0.924	0.692
1.				0.645		0.434	0.926	0.479
21.				0.619		0.676	0.922	0.752
14.					0.756	0.545	0.924	0.692
35.					0.729	0.613	0.924	0.699
13.					0.640	0.558	0.924	0.591
Cronbach’s alfa	0.927	0. 847	0.851	0.802	0.773			
Eigenvalues	9.473	2.681	1.789	1.171	1.136			
% Variance explained	39.472	11.173	7.453	4.878	4.735			

h^2^: communalities

**Fig. 1:**
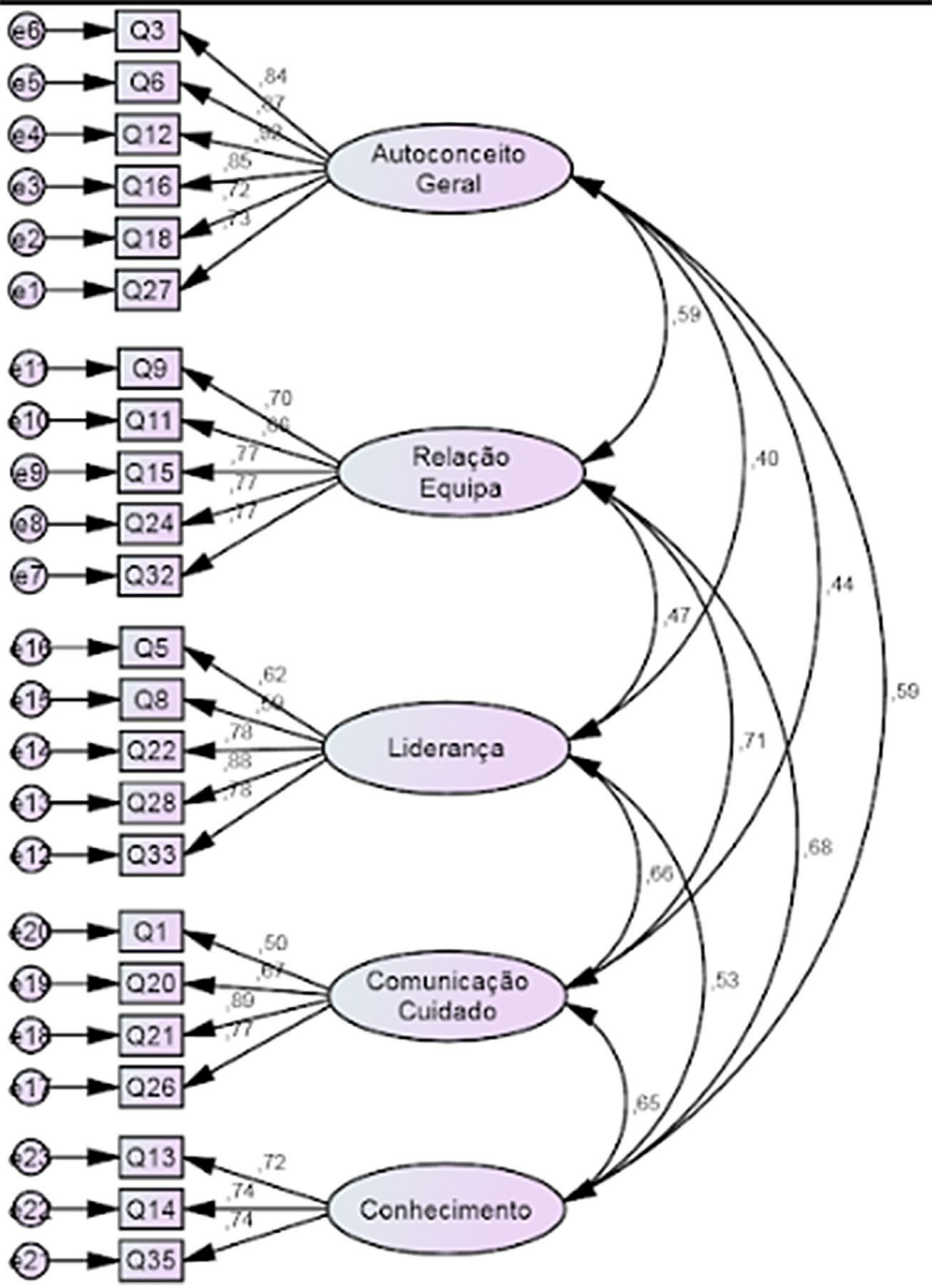
CFA of the Pt – NSCQ. Autoconceito geral – General self-concept; Relação com a equipa – Staff Relations; Liderança – Leadership; Comunicação-cuidar - Care-communication; Conhecimento - Knowledge

The model fit was tested through CFA and revealed a good fit. Overall, the statistics were within acceptable ranges, namely: CFI = 0.937, the χ2/df = 1.81, RMSEA = 0.061, RMR = 0.042. The only exception was GFI = 0.865, however it is near the acceptable value (Fig. 1). The total α of the instrument was 0.927.

Pearson’s correlation coefficient was used to analyse the correlations between the dimensions of the Pt – NSCQ, and all the factors correlate with each other. The observed correlations are moderate to strong, positive, and statistically significant (Table 2).

**Table 2:** Correlations between Pt – NSCQ dimensions

		General self-concept	Staff relations	Leadership	Care-communication	Knowledge
General self-concept	Pearson correlation	1				
	Sig. (bilateral)					
Staff relations	Pearson correlation	0.674	1			
	Sig. (bilateral)	0.000*				
Leadership	Pearson correlation	0.567	0.629	1		
	Sig. (bilateral)	0.000*	0.000*			
Care-communication	Pearson correlation	0.452	0.723	0.721	1	
	Sig. (bilateral)	0.000*	0.000*	0.000*		
Knowledge	Pearson correlation	0.614	0.683	0.636	0.624	1
	Sig. (bilateral)	0.000*	0.000*	0.000*	0.000*	

*Note* *The correlation is significant at the 0.01 level (bilateral)

## Discussion

Professional self-concept has an impact on academic performance and influences students’ future professional practice. In fact, in a recent study [43], emphasised the importance of intervening and directing attention to the development of professional self-concept in this population.

The validity and reliability of an instrument depend on a systematic translation process and cultural adaptation. In the Turkish validation, a four-step methodology — translation, back-translation, reconciliation, and comparison — was employed, followed by a content validity assessment conducted by ten experts [16]. The studies in China and Taiwan followed a different approach to the Turkish, where in a three-step method was employed with translation, back-translation, and monolingual testing, assessing content validity with eight experts [32, 33].

Notably, none of the mentioned studies conducted a pre-test, except for the current study. A pre-test of the final instrument version was conducted to evaluate the tool, test the data collection platform, and identify potential issues, such as comprehension challenges among the target population. On a similar positive note, the pre-test results showed no missing answers, indicating a good understanding of the items by the participants, aligning with Beaton et al. perspective on effective comprehension and interpretation [26].

In the European Portuguese translation, after the first Delphi panel round, a consensus of 4.67 out of 5 led to changing the Likert-type scale from eight to six points, supported by the author of the original instrument. This change of reducing the response options is considered to maintain the instrument reliability and increases response rates [44]. Holtom et al. highlighted a strong association between questionnaire size and measurement error [45]. Adapting the NSCQ to a shorter version with more objective answers could enhance adherence, response rates, and result validity.

The data analysed came from a national-wide non-probabilistic snowball sample of 216 nursing students from 12 public and private schools. The sample was mostly female (82.40%), in line with the proportion of female nurses registered (82.50%) in the Portuguese Nursing Board [46].

The EFA identified five factors, which are aligned with the empirical structures of those found in the Taiwanese [33] and Turkish [16] studies.

However, the specific factors were not the same. The validation to the Taiwanese context [33] concealed the Leadership dimension, arguing that nursing undergraduate students did not develop these competences. The validation to the Turkish context [16] combined the dimensions of staff relations with communication. In the Pt – NSCQ, the communication dimension was similarly merged yet with another dimension, in this case Care. However, the factor analysis presented in the validation study for Taiwan [33], showed a similar behaviour to the validation carried out in this study, since the “care” dimension retained only three items (1, 20 and 23), with the rest migrating to the “communication” dimension [33]. Also in the validation for Turkey [16], communication” dimension merged with the “staff” dimension. Despite this aggregation of dimensions, the internal consistency of the sub-scale is considered to be good (0.80), similar to the original instrument and the other validations. This aggregation may mean that the participants did not distinguish between the concepts of communication and care, considering them to be similar or integral.

It is important to note that in other instruments that aim to measure professional self-concept in nursing, the communication dimension has been deliberately omitted. In short, integrating the two dimensions into a single one is acceptable because communication is an essential component of providing quality nursing care. Effective communication helps nurses to build trust, understand patients’ needs, provide emotional support, educate patients, and work effectively as part of a healthcare team.

To improve interpretability and psychometric properties, 12 items were excluded, resulting in 67.71% of the explained variance, which surpasses other validations. As for the psychometric properties, the total Cronbach’s Alpha of the Pt – NSCQ (0.927) is considered very good and comparable to other versions, as well as the alphas by dimension, which ranged from 0.77 in the knowledge dimension to 0.92 in the general self-concept dimension.

Despite GFI being at the threshold of standard values, altogether, this study has made it possible to obtain an instrument supported by a robust theoretical model, as replicated by the CFA, which will allow to measure and evaluate Portuguese nursing students’ professional self-concept in a valid and reliable manner. Altogether, this study has made it possible to obtain an instrument supported by a robust theoretical model, as replicated by the CFA, which will allow to measure and evaluate Portuguese nursing students’ professional self-concept in a valid and reliable manner.

## Limitations and suggestions

Study limitations might be considered with regards to the small pre-test sample size and the absence of participant interviews to assess comprehension. Future research should incorporate a test-retest method to evaluate the assessment instrument’s temporal stability. It should also be added that no detailed cross-analysis was carried out by year of degree or at different levels of study programme. This study was based on classical test theory. Future studies could consider analysing psychometric properties using item response theory. We also suggest replicating the studies with nurses.

## Conclusions

The process of translation, cultural adaptation, and validation of the Pt – NSCQ revealed structural changes in relation to the original instrument, while yielding adequate psychometric characteristics for the Portuguese population of nursing students. Future studies might consider assessing the psychometric properties with Portuguese students to explore potential heterogeneity of variation of the constructs in relation to the expected evolving professional self-concept.

## Electronic supplementary material

Below is the link to the electronic supplementary material.

Supplementary Material 1

## Data Availability

The datasets used and/or analyzed during the current study are available from the corresponding author upon reasonable request. The data are not publicly available because this issue was not considered within the informed consent signed by the participants of the study.

## Abbreviations

CFA: Confirmatory factor analysis
CFI: Comparative fit Iidex
EFA: Exploratory factor analysis
GFI: Goodness of fit index
NSCQ: Nurse Self-Concept Questionnaire
Pt – NSCQ: *“Questionário de Autoconceito dos/as Enfermeiros/as”*
RMR: Root mean square residual
RMSEA: Root mean squared error of approximation
