# Correction: Translation, cultural adaptation, and validation of the Nurse Self-Concept Questionnaire (NSCQ) for Portuguese nursing students

**DOI:** 10.1186/s12912-024-02422-w

**Published:** 2024-10-11

**Authors:** Inês F. Almeida, Rafael A. Bernardes, Liliana B. Sousa, Paulo Santos-Costa, Filipa Ventura, Amorim Rosa

**Affiliations:** 1 Health Sciences Research Unit: Nursing, Nursing School of Coimbra, Coimbra, Portugal; 2 The Nursing Research, Innovation and Development Centre of Lisbon (CIDNUR), Nursing School of Lisbon (ESEL), Lisboa, Portugal

**Correction: BMC Nursing (2024) 23:422**

10.1186/s12912-024-02031-7

The original article mistakenly omits a Funding statement that the authors wish to note ahead:

‘This work is funded by National Funds through the FCT - Foundation for Science and Technology, I.P., within the scope of the project Ref. UIDB//00742/2020.’

