# Supplementary material for: Translation, cultural adaptation, and validation of the Nurse Self-Concept Questionnaire (NSCQ) for Portuguese nursing students
Source: BMC Nurs. 2024 Jun 21;23:422. doi: 10.1186/s12912-024-02031-7 (PMC11191236; doi:10.1186/s12912-024-02031-7)
Supplement: Supplementary file 1 — Supplementary Material 1 [file 12912_2024_2031_MOESM1_ESM.pdf]

Supplementary file 1 (English version of the Pt-NSCQ)

	1- Definitely false	2-False	3- False most of the time	4- True most of the time	5- True	6- Definitely true
1- I have the necessary skills to respond to my clients' needs						
2- I feel quite satisfied being a nurse						
3- I feel quite satisfied being a nurse						
4- Being a nurse brings me great satisfaction						
5- I'm respected for my leadership skills						
6- I feel satisfied with the professional relationships I have with colleagues						
7- I can relate easily to my colleagues						
8- I like being a nurse						

9- I enjoy communicating information and sharing ideas with colleagues and users						
10- I'm looking for new training opportunities to improve my knowledge of nursing						
11- I get on well with other health professionals						
12- I'm proud to be a nurse						
13- I'm enthusiastic about nursing						
14- Caring for users is easy for me						
15- I can communicate confidently with users and colleagues						
16- I enjoy taking on leadership responsibilities in nursing						

17- I have a good working relationship with other health professionals						
18- Communicating effectively with users and colleagues is easy for me						
19- My work as a nurse is extremely interesting						
20- I assume leadership roles with confidence						
21- I hope I can continue to look after users						
22- I'm able to establish good working relationships with other healthcare professionals						
23- Being a good leader in nursing is easy for me						

24- I enjoy acquiring new knowledge in nursing						
